# The Physical Treatment of Insomnia

**Published:** 1900-07-07

**Authors:** C. J. Whitby


### Hospital Clinics and Medical Progress.

### THE PHYSICAL TREATMENT OF INSOMNIA.

By C. J. Whitby, B.A., M.D.Cantab.

The use of hypnotics upon emergency will always be justifiable, but for the treatment of habitual sleeplessness they have many disadvantages. A conscientious effort, which will often succeed, should therefore be made to obtain the desired effect by other means. The direct "repressive" treatment of symptoms is seldom a satisfactory procedure, and the administration of medicinal hypnotics is no exception to the rule. One of the earliest results is the establishment in the mind of the sufferer of the fixed idea that natural sleep is "past praying for"; and this very conviction forms an almost insurmountable barrier to recovery.[1] On the other hand, it is surprising what simple expedients will, at the expense of a little preliminary consideration of the cause of the functional failure, sometimes prove successful beyond our hopes. From a clinical point of view, cases of insomnia may, I think, be divided into four main groups, and though it will, no doubt, often happen that a particular case presents features common to several groups, those of one or the other will usually predominate.

1. **Reflex Type.**—A large number of cases of insomnia are produced solely by dyspepsia, and especially by that form of gastro-enteric derangement which results in fermentative putrefaction of the ingesta. It is typical of this class that the patient sleeps well for an hour or two, and then wakes, usually with a start, to find himself the subject of an attack of palpitation. In milder cases it may be obvious to the physician that the failure to sleep is due to morbid nerve influences of gastric or enteric origin, although, not improbably, the patient may emphatically disclaim the existence of "indigestion." The careful regulation of his diet, and, more especially, the avoidance of an ill-timed or unsuitable supper (*e.g.*, one containing mechanically-irritating or easily fermentable materials) will not always suffice in these cases to break the dyspeptic habit and restore sleep. Dietetic measures should then be reinforced by the administration at bed time of a well-applied hot abdominal fomentation, replaced in 20 minutes by a swansdown calico compress, four-fold, measuring 8 in. by 6 in., wrung out of very hot water, and applied to the epigastrium, covered by a piece of waterproof and secured by a broad flannel waist-bandage. The cumulative sedative action of the constant warmth and moisture is reflected from the cutaneous nerve-endings through the spinal cord and the solar plexus to the visceral branches of the sympathetic; and at the same time visceral, and, probably also, cerebral congestion is relieved by the derivative effect of the application. In asthenic individuals and in cold weather a poultice may be preferable. Flatulency is often relieved by a hot drink. Dyspeptics who are bad sleepers should therefore have at their bedside a simple heating apparatus containing some suitable liquid—not tea or coffee. Fæcal accumulations is another possible cause of reflex insomnia; and I recently noticed in a case of menorrhagia that several restless nights were followed by the extrusion of some large clots per vaginam. Uterine irritation may be allayed by sitz baths of from 10 to 15 minutes duration, at a temperature of 80 deg. F.

2. **Circulatory Type.**—In many instances insomnia is apparently a result of irregular or defective circulation of cardiac or vaso-motor origin, or both, and the majority of these cases can, I think, be cured without internal medication. A typical example is the reading man of sedentary habit, who goes to bed late after an evening spent in severe mental effort, and is compelled by the dilated arterioles of his cerebral hemispheres, and the functional activity they involve, to go on wrestling with unsolved problems for weary hours at great expense to health and temper. The therapeutic key to the above situation may be found in the following generalisations[2]: (1) "There are probably few exceptions to the rule (verified experimentally by Schlikoff and Winternitz) that local contraction or dilatation of arteroides, however induced, is accompanied and followed by contrary behaviour on the part of vessels elsewhere." (2) "There are probably many distinct parts of the organism which have positive or negative neural or vaso-motorial relations to each other—for example, the feet and the head, and, according to Winternitz, the hands and the organs of respiration." The first rule in treating this type of insomnia should be to ensure the continued vital, not merely passive, warmth of the feet during the night, and this cannot be effected by the mere approximation of a jar of hot water placed under the bedclothes. Peterson[3] states that by footbaths of cold running water the temperature in the external auditory meatus can be reduced by one degree, and that meanwhile the conjunctival vessels may be seen to contract. Probably there is a simultaneous contraction of the vessels of the pia mater, as this may be produced experimentally in rabbits by thermal procedures, which produce dilatation of vessels in other parts of the body. The plan I suggest is as follows: The patient should keep his feet in water as hot as he can comfortably bear it for ten minutes. He should then put on a pair of thin cotton socks wrung out of cold water, and over these a somewhat larger pair of warm woollen ones. A hot-water jar is rarely necessary, and if used should be retained only till reaction has fairly begun. The advantage of this plan is that if a patient once gets off to sleep he rarely wakes before daylight. A more powerful derivative, and, consequently, hypnotic, effect is producible by a mustard sitz bath, a simple procedure enough, but well worth a trial in obstinate cases. The temperature of the water should be about 105 deg. F., and its depth sufficient just to cover the groins of the patient. The strength of the decoction should be a breakfast-cupful of mustard bran to the gallon of hot water. Duration, 20 minutes, or less if felt too acutely. In cases where patients complain of cold feet, Dr. Lewis Jones strongly recommends the electric footbath. An induction coil is used, and the wires are attached to two metallic plates, which are placed at the two ends of an ordinary earthenware footbath filled with warm water. The patient is instructed to use this bath at bedtime for ten or fifteen minutes, the current being used as strong as is comfortably bearable. I have frequently prescribed a practically identical bath myself during the past ten years, and can confirm Dr. Jones' statement that "the electric stimulation seems to improve the circulation in the extremities to an extent far superior to anything which can be obtained from an ordinary warm footbath." In some cases, however, an exciting rather than a sedative effect will be the immediate result of such a bath, and it may then be taken in the afternoon.

3. **Toxæmic Type.**—This clinical group consists of cases characterised by cutaneous hyperæsthesia, frequently of lithæmic or alcoholic origin, manifesting itself by restlessness and general irritability. This condition can be combated with more or less success by one or other of three physical procedures: Wet packing (hot or cold), warm reclining baths, and massage. For maniacal insomnia, Peterson strongly recommends hot packs, and perhaps also, in milder cases, they are of more general utility than cold ones. Still, in the somewhat analogous case of the insomnia and restlessness of influenza, the calming and sedative effect of a cold pack of an hour's duration is often remarkable. When the reactive power is deficient, cold packing may be usefully preceded by 30 minutes massage. Massage for an hour or so, given at or near bedtime, is often prescribed for sleeplessness, but it sometimes has an exciting effect, for a time at least, and should then be administered earlier. The more violent movements and manipulations are better omitted. The narcotic effect of reclining baths of indifferent temperature (98 to 100 F.) is pretty generally recognised, and finds appropriate application in this class of cases. The result is probably due to the "damping" of the sensory nerve-endings by imbibed water; the baths should therefore be of at least 15 minutes' duration. Should the above measures fail, a course of hot-air or vapour baths, or the combination of hot douching and massage known as the Aix douche, may be successful. Such procedures are especially indicated in cases characterised by a high-tension pulse (not due to arterio-sclerosis), and by the frequent deposition of lithatis in excess.

4. **Neurotic Type.**—In a certain percentage of cases the sleeplessness appears to be due to a primary defect of cerebral function. There is often a strongly neurotic family history; and a tendency to phosphaturia is not uncommon. Such cases are frequently complicated by dyspeptic symptoms, cardiac debility and irregular action of the vaso-motor nerves. The treatment recommended for groups 1 and 2 may therefore be tried with some hope of benefit, particularly the mustard sitz-bath and the faradic foot-bath. Dr. Hedley[4] reports a very successful issue in such a case as the result of a course of general constant current baths of gradually increasing strength (50 to 150 ma.) alternating with the direct application of weak currents to the head and spine, and varied by occasional general faradic baths. By seven weeks of such treatment sleep was practically restored. Electrical treatment of some kind presents perhaps the best chance of restoration, but it must be admitted that these cases are peculiarly obstinate, and, sooner or later, the resort to hypnotics is often indispensable. It is, however, remarkable with how little sleep some patients manage to subsist for years without any obvious deterioration of mind or body.

[1] This symptom might rationally be considered an indication for the trial of treatment by "suggestion." [2] Brit. Med. Jour., Dec. 8, 1894, p. 1,305. [3] Amer. Jour. Med. Sci., Feb., 1893. [4] Hydro-Electric Methods, London, 1896, pp. 45-50.

### SURGERY IN PHTHISIS.

It can hardly be a matter for surprise that among the various methods put forward for the cure of pulmonary tuberculosis surgical proceedings should take a share. Everywhere else the complete removal of tuberculous deposits has seemed to be one of the most important steps towards recovery, and although the dead-house appearances in the lungs of cases dying from phthisis are generally such as to show the complete hopelessness of surgical intervention in the later stages of the disease, the conditions found in the post-mortem examination of patients known to be consumptive, but dying from other causes, is now and then such as to suggest that if only surgery could but have stepped in something might possibly have been done.

We have before us two recent contributions to the medical journals dealing with this subject from two quite opposite points of view. One by Mr. Foster Palmer,[1] president of the Chelsea Clinical Society, and the other by Dr. Sherman Leach,[2] of Columbus, U.S.A. Mr. Palmer enters into a general review of the possibility of extirpating the disease by operation, and as the result of his inquiries he concludes that the three principal dangers to be guarded against in operations on the lungs are pneumothorax, surgical emphysema, and hæmorrhage. He lays especial stress on the first of these conditions, and points out that to avoid this danger one or other of the following methods are at our disposal: (1) to sew the pulmonary to the costal pleura, beyond the seat of the disease, before operating; (2) to trust, in certain cases, to the existence of adhesions already formed; (3) to excite artificial adhesions in the pleura; and (4) to expand the lungs artificially from within.

Take it altogether, however, and notwithstanding a few remarkable cases which have been published, we venture to doubt whether it can often be possible to make so sure of the disease being distinctly localised to a removable portion of the lung as to make it practical to put in force the general dictum which seems to be the basis of Mr. Palmer's thesis, that as the only certain cure is extirpation, extirpation ought to be performed.

Hunter's other dictum that short of complete extirpation "cutting always does harm," is equally worthy of consideration. Here and there no doubt cases are met with in which complete collapse and cicatrisation of large cavities appears to be hindered by rigidity of the chest walls, and in such cases operation may be serviceable, much in the same way as operation not infrequently renders possible the closing of an old empyæma, which otherwise would have remained fistulous. But that is a very different thing from proposing excision of tuberculosis of the lung, especially as the proposal is made only in regard to cases in which the patient "feels comparatively well," and suffers only from a "small patch of tubercle"—the very cases in which one can almost promise a cure if the patient can but give himself up to it.

Dr. Leach, however, has a very different proposal. He devotes his remarks to an enforcement of the advantages of a mode of treatment which was first suggested, we believe, by Murphy, of Chicago—namely, the artificial collapsing of the affected lung by means of nitrogen gas injected into the pleura. The idea was originally derived from the observation, which has been made independently by many physicians, that the occurrence of extensive pleuritic effusion has seemed in some cases to be followed by great improvement in the general phthisical symptoms; and it is now held by those who advocate this line of treatment that by its means not only may we hope to place the affected lung at rest, but we may block off the various avenues by which the infection is disseminated or transmitted to other parts of the lung, namely, the bronchi and air vesicles, and the lymphatic circulation. Especial importance is attributed to the latter.

Regarding the operation, the special apparatus required consists of a suitable tank for storing the gas, a graduated bottle in which it may be measured, and a third bottle for securing the hydrostatic pressure, by means of which the gas may be forced into the pleural cavity. The skin of the chest must be thoroughly cleaned with soap and water, and then with alcohol. The water in the bottle must be sterilised, as also the trocar. The point at which the trocar should be entered is given as the fifth or sixth interspace. It is important before the gas is turned on to know that the needle is in the pleural cavity, and not in the lung. "This, however," it is said, "is not difficult to determine," but by what means we are not told. The amount of gas required seems to vary greatly, namely, from 60 to 200 cubic inches, the amount to be given in each case being regulated by the symptoms produced—namely, pain, distress, laboured breathing, extreme cyanosis, rapid heart's action, displacement of the diaphragm. "The respiratory sounds on that side should be absolutely suppressed, unless some of the symptoms render it necessary to stop short of obtaining that point."

Comparing the two lines of thought which have led to the two lines of treatment suggested in the papers we have referred to, we cannot but be struck by the facts, first, that the measures proposed are themselves severe, and by no means devoid of serious risks; second, that the whole subject is at present in the theoretical stage; and finally, that they are mutually contradictory, for while the one observer shows in how large a proportion of cases we can count upon being assisted by the presence of adhesions between the opposed surfaces of the pleura, the success of the other plan would seem to hinge largely upon the absence of any such bands as would oppose the free separation of the visceral and the costal layer.

[1] Lancet, June 23. [2] Columbus Med. Jour., May.

